# The clinical outcomes and tolerability of adding radiotherapy to first-line chemoimmunotherapy for treating patients with metastatic or relapsed esophageal squamous cell carcinoma: A retrospective cohort study

**DOI:** 10.12669/pjms.41.9.12491

**Published:** 2025-09

**Authors:** Xin Hou, Zhen Ren, Rui Duan, Xin Ding

**Affiliations:** 1 Xin Hou Department of Radiation Oncology, Affiliated Hospital of Xuzhou Medical University, Xuzhou, China; 2 Zhen Ren Department of Radiation Oncology, Affiliated Hospital of Xuzhou Medical University, Xuzhou, China; 3 Rui Duan Department of Radiation Oncology, Affiliated Hospital of Xuzhou Medical University, Xuzhou, China; 4 Xin Ding Department of Radiation Oncology, Affiliated Hospital of Xuzhou Medical University, Xuzhou, China

**Keywords:** Chemoimmunotherapy, Esophageal squamous cell carcinoma, Immunotherapy, Radiotherapy

## Abstract

**Objective:**

This investigation sought to assess the clinical outcomes and safety of adding radiotherapy (RT) to first-line chemoimmunotherapy (CIT) for advanced esophageal squamous cell carcinoma (ESCC).

**Methodology:**

In this retrospective cohort study, patients with advanced ESCC (including metastatic or relapsed disease) who received first-line CIT at the Affiliated Hospital of Xuzhou Medical University between 2020 and 2023 were enrolled. Patients were categorized into the CIT alone and CIT plus RT (CI-RT) cohorts. Clinical outcomes and adverse events (AEs) were compared between the two cohorts.

**Results:**

This study enrolled 141 patients (CI-RT, n = 75; CIT, n = 66). The median follow-up duration was 31.7 months (95% confidence interval (CI): 24.5-38.9). The CI-RT cohort had significantly longer median PFS (16.2 vs. 9.3 months, hazard ratio (HR) = 0.652, *P* = 0.022) and median OS (25.2 vs. 14.6 months, HR = 0.591, *P* = 0.012) than did the CIT cohort. Multivariate analysis identified CI-RT as an independent predictor of both PFS (HR = 0.61, 95% CI: 0.42-0.89) and OS (HR = 0.57, 95% CI: 0.38-0.88). The incidence of grade ≥3 AEs did not significantly differ between the two cohorts (*P* = 0.810). Patients in the CI-RT cohort were associated with a higher incidence of esophagitis than were patients in the CIT cohort (16.0% vs. 1.5%, *P* = 0.003). One patient in the CIT cohort died from treatment-related pneumonitis.

**Conclusions:**

When first-line CIT is used for patients with advanced ESCC, the addition of RT can improve patient prognosis while maintaining favorable safety profiles.

## INTRODUCTION

Esophageal cancer (EC) ranks 11th among malignant tumors in terms of global incidence and 7th in terms of mortality^1^ and remains a major health challenge worldwide. Esophageal adenocarcinoma predominates in Europe and the United States, whereas esophageal squamous cell carcinoma (ESCC) is more common in Southeast Asia, including China.^2^ Approximately 40-50% of cases of EC are diagnosed at advanced stages,^3^,^4^ and despite comprehensive radical treatment with chemoradiotherapy/surgery, 40-50% of patients still experience recurrence.^5^,^6^

The development of checkpoint inhibitor therapies has brought hope to patients with advanced EC in recent years, and several clinical studies^7^-^9^ have confirmed that first-line chemoimmunotherapy (CIT) can lead to improved survival. However, these benefits remain limited, with median progression-free survival (mPFS) durations of 6.3-7.3 months and median overall survival (mOS) durations not exceeding 18 months, and are not yet able to meet the enormous clinical treatment needs. Thus, there is a critical need to develop new treatment modalities to further improve the prognosis of these patients.

As an important local treatment for advanced EC, radiotherapy (RT) effectively alleviates dysphagia and pain, improving patients’ quality of life. In the era of immunotherapy, whether RT can act as a potential modality for combination therapy and provide enhanced survival benefits for patients with advanced EC has attracted great attention from oncologists.

Since 2022, although several studies^10^-^12^ have investigated the therapeutic effects of CIT plus RT (CI-RT) for patients with advanced EC, the conclusions of these studies have been inconsistent, and the value of this combination remains controversial. Moreover, the optimal timing for RT intervention in immunotherapy remains undefined. Therefore, we conducted this retrospective study to provide new evidence regarding these questions.

## METHODOLOGY

Data from patients with metastatic or recurrent ESCC treated at our institution from 2020 to 2023 were retrospectively examined. The patients were categorized into CIT alone and CI-RT cohorts based on whether they had undergone RT. Eligibility required fulfillment of the following conditions: histopathological diagnosis of ESCC; confirmed diagnosis of metastatic ESCC (metastasis to nonregional lymph nodes (LNs) and/or distant organs at initial diagnosis) according to the 8^th^ Edition AJCC Cancer Staging Manual or recurrent ESCC (local recurrence and/or distant metastasis occurring ≥6 months after comprehensive radical treatment with chemoradiotherapy/surgery), excluding patients with only local recurrence; treatment with first-line CIT, either alone or in combination with RT; RT administered within three months following the last cycle of immunotherapy; and Eastern Cooperative Oncology Group (ECOG) score ≤1.

### Ethical Approval:

The ethics committee of the Affiliated Hospital of Xuzhou Medical University approved this study (Approval number: XYFY2024-KL440-01; date: September 12, 2024), and the requirement for informed consent was waived due to the noninterventional design of the study.

### Exclusion criteria:

- Patients with other malignant tumors or autoimmune diseases.
- Patients with incomplete medical records.

### Treatment:

CIT was delivered triweekly to all enrolled patients, with treatment being sustained in the absence of disease progression or intolerable toxicity. The chemotherapy regimens included paclitaxel- or fluorouracil-based chemotherapy, administered with or without cisplatin. Paclitaxel (175 mg/m²) was intravenously infused on Day one; fluorouracil (750 mg/m²) was given as a continuous intravenous infusion from Day one to Day five; and cisplatin (75 mg/m²) was administered via intravenous infusion on Day one. Immunotherapy drugs included camrelizumab, tislelizumab, sintilimab, and pembrolizumab. These agents are administered intravenously at a dose of 200 mg on Day one of the treatment cycle. RT and immunotherapy were administered either concurrently or sequentially, and RT was delivered using six MV X-rays to irradiate the involved area via intensity-modulated radiation therapy (IMRT) techniques. In the CI-RT cohort, patients with metastatic ESCC and local recurrence underwent RT targeting the primary esophageal lesion or locally recurrent lesion, as well as nonregional LNs that could be contained within the same radiation field. The need for RT targeting additional metastatic lesions was determined by the treating physician based on each patient’s symptoms and associated risks.

### Outcome assessment and follow-up:

Oligometastasis was defined as the presence of ≤5 metastatic lesions and the involvement of ≤3 metastatic organs. Metastatic lesions were categorized as either nonregional LN metastases or organ metastases. Lesions within the same region were counted as a single lesion. Locoregional recurrences included anastomotic/tumor bed recurrences, regional LN recurrences, and esophageal recurrences. The immunotherapy-to-RT interval (IRI), defined as the time between the last immunotherapy administration before RT initiation and the start of radiation treatment. The follow-up period ended on December 31, 2024. The assessment of treatment-related adverse events (AEs) followed the standardized guidelines in the Common Terminology Criteria for Adverse Events version 5.0 (CTCAE v5.0).

### Statistical analysis:

The primary endpoints were PFS and OS. PFS duration was calculated from immunotherapy commencement until the earliest occurrence of radiologically confirmed disease progression or mortality. The OS interval spanned treatment initiation to either patient death or the final documented follow-up encounter. Baseline characteristics and differences in toxicity incidence were compared using chi-square tests or Fisher’s exact tests, as appropriate. Kaplan-Meier analysis was conducted for PFS and OS, and group differences were examined via the log-rank test. Prognostic factors were identified through univariate and multivariate Cox regression analyses. Factors with P<0.1 in the univariate analysis were included in the multivariate Cox analysis. Two-sided tests were used, with *P*<0.05 indicating statistical significance. Statistical analysis was performed using SPSS (version 25.0) or R (version 4.1.3).

## RESULTS

### Patient Baseline Characteristics:

A total of 141 eligible patients were included in the study, with 75 in the CI-RT cohort and 66 in the CIT cohort. The median age of the patients in the analytical cohort was 65 years (interquartile range (IQR): 57-69). The most frequent site of LN metastasis was the supraclavicular LNs (43 patients, 30.5%). The lungs were the most common site of organ metastasis (41 patients, 29.1%) (Supplementary Table-I). Locoregional recurrence occurred in 42 patients, with regional LNs being the most frequent sites (29 patients, 69.0%) (Supplementary Table-II). The median number of immunotherapy cycles in the CI-RT and CIT cohorts was 4 (IQR: 3-9) and 4 (IQR: 3-8), respectively (*P* = 0.761). Similarly, the median number of chemotherapy cycles in the CI-RT and CIT cohorts was 4 (IQR: 3-5) and 4 (IQR: 4-5), respectively (*P* = 0.519). Both cohorts demonstrated comparable clinicopathological profiles at baseline (Table-I). In the CI-RT cohort, 66 patients received conventional fractionated RT with a total dose of 50-60 Gy delivered in daily fractions of 1.8-2.0 Gy, and the median prescribed dose was 50.4 Gy. Additionally, nine patients received hypofractionated RT for metastatic lesions, with a total dose of 30-50 Gy delivered in daily fractions of 3.0-5.0 Gy, and the median dose per fraction was 3 Gy (Supplementary Table-III). The median IRI was one month (IQR: 0.5-2). Eight patients received concurrent immunotherapy and RT, and 67 underwent sequential RT.

**Supplementary Table-I:** The site of metastasis.

Location of metastasis (n = 141)	n (%)
Cervical LNs	31 (22.0)
Supraclavicular LNs	43 (30.5)
Mediastinal nonregional LNs	22 (15.6)
Axillary LNs	8 (5.7)
Abdominal LNs	28 (19.9)
Pelvic LNs	3 (2.1)
Lung	41 (29.1)
Liver	31 (22.0)
Bone	25 (17.7)
Adrenal gland	6 (4.3)
Spleen	1 (0.7)

LNs, Lymph nodes.

**Supplementary Table-II:** The site of locoregional recurrences.

Location of locoregional recurrence^a^(n = 42)	n (%)
Anastomosis/tumor bed	10 (23.8)
Regional LNs	29 (69.0)
Esophagus	9 (21.4)

a:Percentage calculations for locoregional recurrence sites are based on patients with locoregional recurrence; LNs, Lymph nodes.

**Table-I:** Clinicopathologic characteristics of all patients.

Characteristics	Total No. (%)	CI-RT No. (%)	CIT No. (%)	P Value
All	141 (100.0)	75 (53.2)	66 (46.8)	
***Age (years)***				
>65	73 (51.8)	35 (46.7)	38 (57.6)	0.196
≤65	68 (48.2)	40 (53.3)	28 (42.4)	
***Sex***				
Female	31 (22.0)	15 (20.0)	16 (24.2)	0.544
Male	110 (78.0)	60 (80.0)	50 (75.8)	
***Location of primary tumor***				
Lower thoracic	62 (44.0)	36 (48.0)	26 (39.4)	0.588
Middle thoracic	55 (39.0)	27 (36.0)	28 (42.4)	
Upper thoracic	24 (17.0)	12 (16.0)	12 (18.2)	
***Tumor length (cm)***				
>5	83 (58.9)	45 (60.0)	38 (57.6)	0.770
≤5	58 (41.1)	30 (40.0)	28 (42.4)	
***Pathological differentiation***				
High	44 (31.2)	25 (33.3)	19 (28.8)	0.671
Medium	55 (39.0)	30 (40.0)	25 (37.9)	
Low	42 (29.8)	20 (26.7)	22 (33.3)	
***T stage***				
T1-3	103 (73.0)	51 (68.0)	52 (78.8)	0.150
T4	38 (27.0)	24 (32.0)	14 (21.2)	
N stage				
N0-1	88 (62.4)	46 (61.3)	42 (63.6)	0.778
N2-3	53 (37.6)	29 (38.7)	24 (36.4)	
***Cancer type***				
Primary metastatic disease	63 (44.7)	38 (50.7)	25 (37.9)	0.128
Recurrence after radical therapy	78 (55.3)	37 (49.3)	41 (62.1)	
***Tumor burden***				
Multiple metastases	58 (41.1)	28 (48.3)	30 (51.7)	0.328
Oligometastases	83 (58.9)	47 (56.6)	36 (43.4)	

CIT, chemoimmunotherapy; CI-RT, chemoimmunotherapy plus radiotherapy; IQR, interquartile range.

**Supplementary Table-III:** The radiation site and dose for patients treated with hypofractionated RT.

Radiation site (No. of patients)	Dose (targets)
Bone (5)	30Gy/10F
Cervical LNs (1)	45Gy/15F
Adrenal gland (1)	40Gy/10F
Lung (2)	50Gy/10F

RT, radiotherapy; LNs, lymph nodes; F, fraction.

### Survival outcomes:

**Fig.1:**
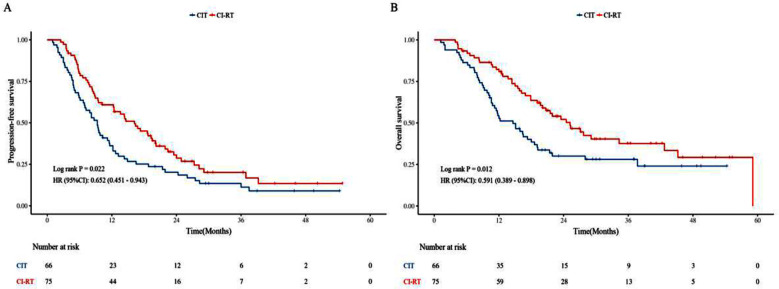
Kaplan-Meier curves of progression-free survival (A) and overall survival (B) in all patients. CIT, chemoimmunotherapy; CI-RT, chemoimmunotherapy plus radiotherapy; HR, hazard ratio; CI, confidence interval.

At the conclusion of the follow-up period, with a median follow-up duration of 31.7 months (95% confidence interval (CI): 24.5-38.9), 90 patients had died. The mPFS of the CI-RT and CIT cohorts was 16.2 (95% CI: 12.30-20.20) and 9.3 (95% CI: 6.90-11.60) months, respectively (*P* = 0.022) (Fig.1A). The mOS was 25.2 (95% CI: 20.10-45.20) months in the CI-RT cohort and 14.6 (95% CI: 10.80-19.10) months in the CIT cohort (*P* = 0.012) (Fig.1B).

### Univariate and multivariate analyses of prognostic factors:

Multivariate Cox regression analysis demonstrated that CI-RT was independently associated with improved PFS (*P* = 0.010) and OS (*P* = 0.010). In contrast, male sex independently predicted poorer outcomes in terms of PFS (*P* = 0.038) and OS (*P* = 0.044) (Table-II).

**Table-II:** Univariate and multivariate analyses for PFS and OS.

Variable	PFS				OS			
	Univariate		Multivariate		Univariate		Multivariate	
	HR (95%CI)	P Value	HR (95%CI)	P Value	HR (95%CI)	P Value	HR (95%CI)	P Value
Age (≤65 vs. >65 years)	1.02 (0.71-1.48)	0.910			0.99 (0.65-1.49)	0.944		
Sex (Male vs. Female)	1.50 (0.95-2.35)	0.082	1.63 (1.03-2.57)	0.038	1.75 (1.01-3.01)	0.045	1.76 (1.01-3.06)	0.044
***Location of primary tumor***								
(Middle vs. Lower)	0.80 (0.54-1.20)	0.278			0.79 (0.50-1.24)	0.309		
(Upper vs. Lower)	0.70 (0.40-1.23)	0.218			0.92 (0.50-1.69)	0.797		
Tumor length (≤5 vs. >5cm)	0.87 (0.60-1.26)	0.464			0.86 (0.57-1.32)	0.495		
***Pathological differentiation***								
(Low vs. High)	1.36 (0.85-2.19)	0.203			1.09 (0.65-1.84)	0.741		
(Medium vs. High)	1.08 (0.69-1.69)	0.733			0.85 (0.51-1.41)	0.534		
T stage (T4 vs. T1-3)	1.12 (0.74-1.69)	0.589			1.07 (0.68-1.71)	0.762		
N stage (N2-3 vs. N0-1)	0.96 (0.66-1.41)	0.842			0.97 (0.63-1.50)	0.896		
***Cancer type***								
(Recurrence vs. Primary metastatic disease)	1.01 (0.70-1.46)	0.973			0.85 (0.56-1.28)	0.431		
***Tumor burden***								
(Oligometastases vs. Multiple metastases)	0.82 (0.56-1.19)	0.295			0.62 (0.41-0.94)	0.024	0.69 (0.45-1.05)	0.084
Treatment modality (CI-RT vs. CIT)	0.65 (0.45-0.94)	0.023	0.61 (0.42-0.89)	0.010	0.59 (0.39-0.90)	0.014	0.57 (0.38-0.88)	0.010

PFS, progression-free survival; OS, overall survival; HR, hazard ratio; CI, confidence interval; CIT, chemoimmunotherapy; CI-RT, chemoimmunotherapy plus radiotherapy.

### Subgroup analyses:

**Fig.2:**
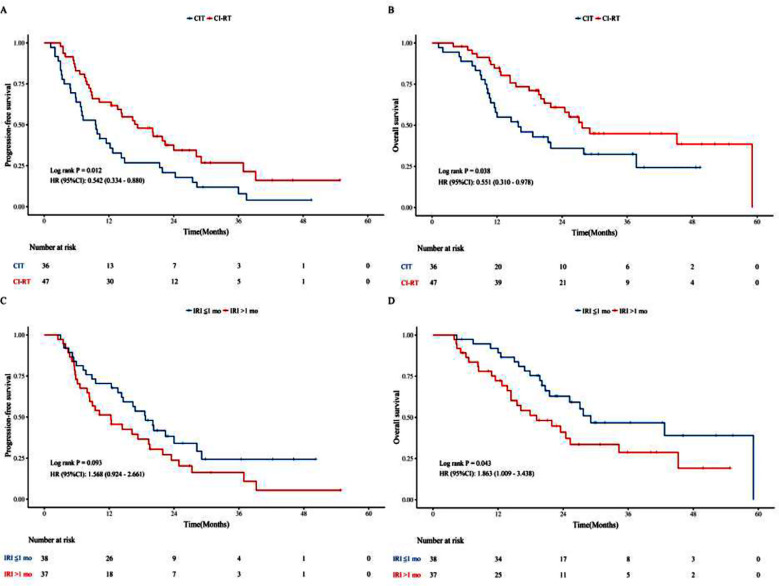
Kaplan-Meier curves of PFS (A) and OS (B) in patients with oligometastases. Kaplan-Meier curves of PFS (C) and OS (D) between the “IRI ≤1 month” and “IRI >1 month” groups. PFS, progression-free survival; OS, overall survival; CIT, chemoimmunotherapy; CI-RT, chemoimmunotherapy plus radiotherapy; HR, hazard ratio; CI, confidence interval; IRI, immunotherapy-to-radiotherapy interval; mo, month.

**Supplementary Fig.1:**
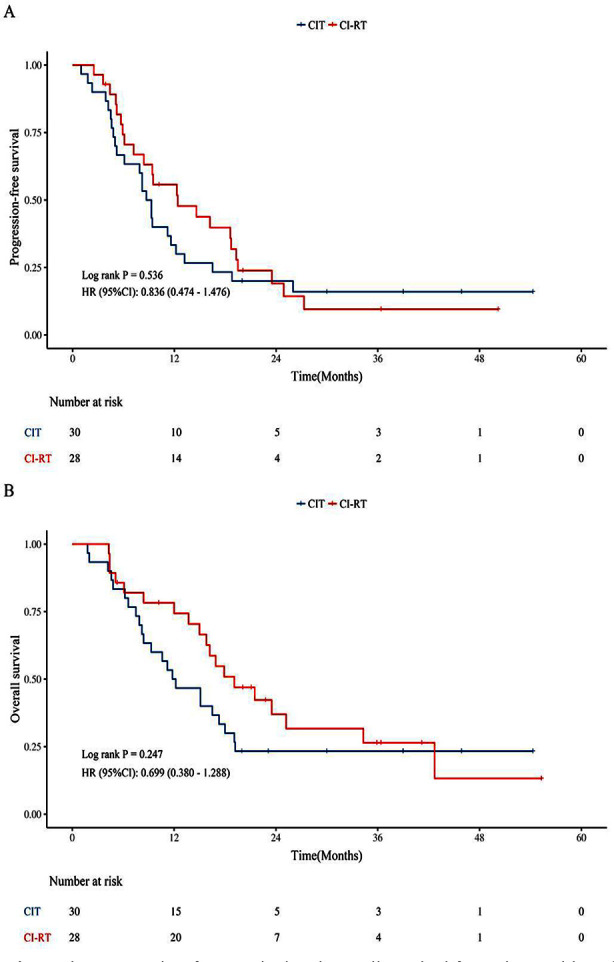
The progression-free survival and overall survival for patients with multiple metastases. CIT, chemoimmunotherapy; CI-RT, chemoimmunotherapy plus radiotherapy; HR, hazard ratio; CI, confidence interval.

We conducted subgroup analyses of patients with oligometastases and multiple metastases. In the oligometastases group, CI-RT significantly improved the mPFS (17.3 vs. 9.6 months, *P* = 0.012) and mOS (27.7 vs. 15.9 months, *P* = 0.038) compared with CIT alone (Fig. 2A and B). However, in the Multiple metastases group, CI-RT did not result in better mPFS (12.4 vs. 9.0 months, *P* = 0.536) or mOS (19.1 vs. 12.0 months, *P* = 0.247) compared with CIT alone (Supplementary Fig.1A and B).

**Supplementary Fig.2:**
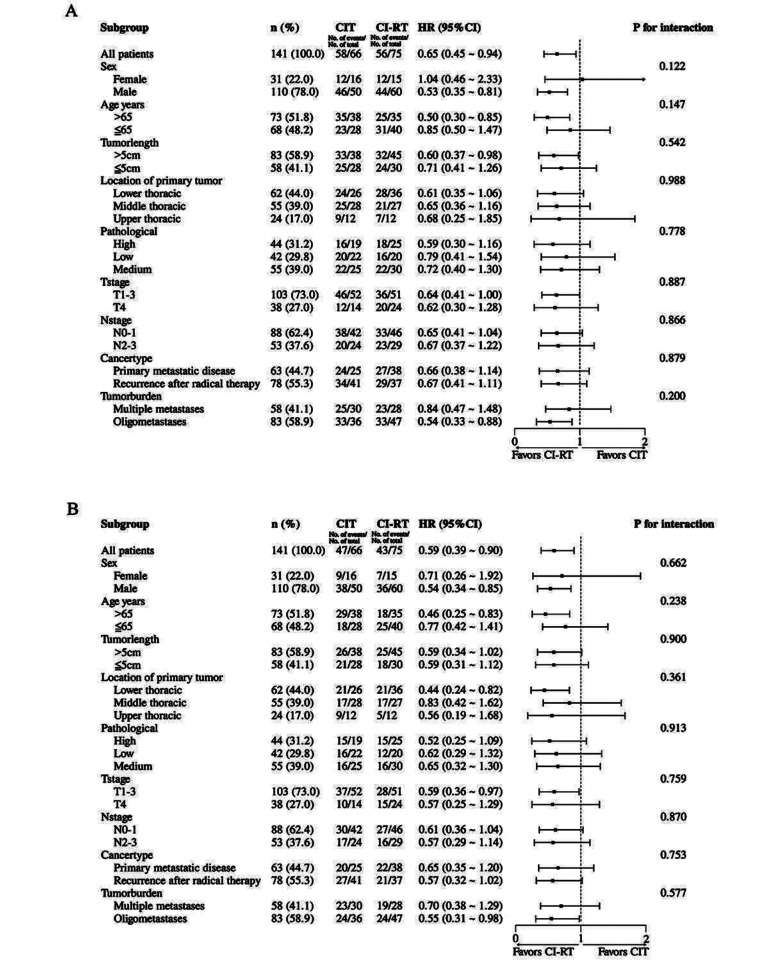
Forest plot of subgroup analyses for progression-free survival (A) and overall survival (B). CIT, chemoimmunotherapy; CI-RT, chemoimmunotherapy plus radiotherapy; HR, hazard ratio; CI, confidence interval.

To investigate the impact of IRI on therapeutic outcomes, the CI-RT cohort was categorized into two subgroups: the “IRI ≤1 month” (n = 38) and “IRI >1 month” (n = 37) groups. The mPFS of the “IRI ≤1 month” and “IRI >1 month” groups was 18.7 and 12.3 months, respectively (*P* = 0.093) (Fig. 2C). Furthermore, the mOS of the “IRI ≤1 month” and “IRI >1 month” groups was 29.1 and 19.1 months, respectively (*P* = 0.043) (Fig.2D). The *P*-value for interaction was greater than 0.05 in all subgroups, suggesting an absence of statistically significant interaction effects between the treatment modality and the characteristics of each subgroup, as illustrated in Supplementary Fig.2A and B.

### Treatment-related adverse events:

Treatment-related AEs are summarized in Supplementary Table-IV. Sixteen (21.3%) patients in the CI-RT cohort and 13 (19.7%) in the CIT cohort experienced AEs of grade ≥3, with no difference between cohorts (*P* = 0.810). Patients in the CI-RT cohort were associated with a higher incidence of esophagitis than were patients in the CIT cohort (16.0 vs. 1.5%, respectively; *P* = 0.003). One (1.5%) patient in the CIT cohort developed pneumonitis that resulted in death. Moreover, the incidence of AEs did not differ significantly between the two IRI-based groups (≤1 month vs. >1 month) (Supplementary Table-V).

**Supplementary Table-IV:** Treatment-related adverse events Data.

adverse events	Any Grade n (%)		P Value	Grade ≥3 n (%)		P Value
	CI-RT (n = 75)	CIT (n = 66)		CI-RT (n = 75)	CIT (n = 66)	
Leukopenia	26 (34.7)	19 (28.8)	0.455	7 (9.3)	6 (9.1)	0.960
Neutropenia	23 (30.7)	18 (27.3)	0.658	7 (9.3)	6 (9.1)	0.960
Thrombocytopenia	7 (9.3)	5 (7.6)	0.709	1 (1.3)	1 (1.5)	1.000
Anaemia	20 (26.7)	19 (28.8)	0.779	0 (0.0)	1 (1.5)	0.468
Nausea or vomiting	13 (17.3)	14 (21.2)	0.559	0 (0.0)	0 (0.0)	NA
Hepatic impairment	3 (4.0)	2 (3.0)	1.000	0 (0.0)	0 (0.0)	NA
RCCEP	10 (13.3)	7 (10.6)	0.620	0 (0.0)	0 (0.0)	NA
Hypothyroidism	6 (8.0)	6 (9.1)	0.817	0 (0.0)	1 (1.5)	0.468
Esophagitis	12 (16.0)	1 (1.5)	0.003	2 (2.7)	0 (0.0)	0.498
Esophageal fistula	3 (4.0)	2 (3.0)	1.000	3 (4.0)	2 (3.0)	1.000
Pneumonitis	8 (10.7)	3 (4.5)	0.176	1 (1.3)	1 (1.5)	1.000
Myocarditis	2 (2.7)	1 (1.5)	1.000	1 (1.3)	0 (0.0)	1.000

CIT, chemoimmunotherapy; CI-RT, chemoimmunotherapy plus radiotherapy;

NA, not available; RCCEP, reactive cutaneous capillary endothelial proliferation.

**Supplementary Table-V:** Treatment-related adverse events in the “IRI ≤1 mo” and “IRI >1 mo” groups.

adverse events	Any Grade n (%)		P Value	Grade ≥3 n (%)		P Value
	IRI ≤1 mo (n = 38)	IRI >1 mo (n = 37)		IRI ≤1 mo (n = 38)	IRI >1 mo (n = 37)	
Leukopenia	14 (36.8)	12 (32.4)	0.688	4 (10.5)	3 (8.1)	1.000
Neutropenia	13 (34.2)	10 (27.0)	0.500	4 (10.5)	3 (8.1)	1.000
Thrombocytopenia	5 (13.2)	2 (5.4)	0.430	1 (2.6)	0 (0.0)	1.000
Anaemia	12 (31.6)	8 (21.6)	0.330	0 (0.0)	0 (0.0)	NA
Nausea or vomiting	6 (15.8)	7 (18.9)	0.720	0 (0.0)	0 (0.0)	NA
Hepatic impairment	2 (5.3)	1 (2.7)	1.000	0 (0.0)	0 (0.0)	NA
RCCEP	6 (15.8)	4 (10.8)	0.768	0 (0.0)	0 (0.0)	NA
Hypothyroidism	2 (5.3)	4 (10.8)	0.430	0 (0.0)	0 (0.0)	NA
Esophagitis	6 (15.8)	6 (16.2)	0.960	1 (2.6)	1 (2.7)	1.000
Esophageal fistula	2 (5.3)	1 (2.7)	1.000	2 (5.3)	1 (2.7)	1.000
Pneumonitis	4 (10.5)	4 (10.8)	1.000	1 (2.6)	0 (0.0)	1.000
Myocarditis	1 (2.6)	1 (2.7)	1.000	0 (0.0)	1 (2.7)	0.493

IRI, immunotherapy-to-radiotherapy interval; mo, month; NA, not available;

RCCEP, reactive cutaneous capillary endothelial proliferation

## DISCUSSION

In this study, we demonstrated that adding RT to first-line CIT for patients with metastatic or recurrent ESCC significantly improved PFS and OS. Subgroup analyses indicated that patients with oligometastases and those with “IRI ≤1 month” may achieve improved survival outcomes from this combination therapy. Furthermore, our study revealed that this combination therapy is safe for clinical application. Our findings have crucial clinical implications by providing real-world evidence to guide optimal RT strategies for patients with advanced ESCC following first-line CIT .

Our study revealed that, compared with patients in the CIT cohort, those in the CI-RT cohort presented significantly superior mPFS and mOS. This survival advantage aligns with the results of the retrospective study by Chen et al.^11^ who analyzed patients with metastatic ESCC who received first-line CIT. Comparative survival outcomes favored the CI-RT approach over CIT alone, with improvements of 3.6 months in mPFS (*P* = 0.002) and 10.3 months in mOS (*P* = 0.003). Furthermore, multivariate analysis demonstrated that RT significantly reduced the risk of death (OS, hazard ratio (HR) = 0.65) and disease progression (PFS, HR = 0.72). Similarly, the AEC-ICR-1st study^13^ found that among patients with advanced ESCC, the combination of RT improved OS in patients receiving first-line CIT compared with CIT alone (21.3 vs. 17.5 months, P = 0.008). These results may be due to the immune-activating effect of radiotherapy.^14^

However, a prior study investigating the combination of immunotherapy with RT in patients with advanced ESCC failed to show significant improvements in OS or PFS in patients receiving RT compared with the control group in the overall population.^10^ Notably, approximately 30% of the cohort received immunotherapy as a second-line therapy only, which may have influenced the study’s conclusions due to treatment heterogeneity. A multicenter retrospective study conducted in China^12^ enrolled 202 patients with metastatic or recurrent ESCC who received pembrolizumab plus chemotherapy as first-line therapy. The results revealed that localized therapy did not significantly improve mOS or mPFS compared with controls. Nevertheless, this study has a limitation in that it does not specify RT details such as dose and timing, which may be potential factors affecting efficacy and could explain the lack of survival difference between the two groups. The prognostic impact of CI-RT in patients with advanced ESCC should be further investigated in well-designed prospective studies.

Oligometastatic disease manifests as a transitional phase in oncological progression, representing an intermediate state between localized disease and systemic metastases.^15^ Our subgroup analysis showed that the survival outcomes associated with CI-RT, including mPFS and mOS, were significantly more favorable in the oligometastases group, but no statistically significant benefit was observed in the multiple metastases group. This finding aligns with previous studies^16^-^18^ on lung, prostate, and colorectal cancers, which demonstrated that combining local therapy with systemic treatment confers a survival advantage over systemic-only regimens in patients with oligometastasis. Another recent study on ESCC also reported similar findings to ours.^19^ Consequently, we propose that for patients with advanced ESCC, treatment strategies should be tailored based on tumor burden, with more aggressive local RT recommended for patients with oligometastasis.

The timing of RT intervention may affect the efficacy of combined RT and immunotherapy, but the optimal timing remains unclear^14^, with limited studies specifically addressing ESCC. In our study, we observed that, compared with that in the “IRI >1 month” group, the mOS in the “IRI ≤1 month” group was significantly improved, and the mPFS was extended by 6.4 months, although no statistically significant difference was detected. This may be due to the limited sample size of patients in the subgroup. Wu et al.^10^ found that, compared with patients with advanced ESCC who received RT outside the three-month window before or after immunotherapy, those who underwent RT within this window exhibited superior mPFS and mOS. This is consistent with our findings, suggesting that a relatively short interval between RT and immunotherapy enhances treatment efficacy. The optimal time windows identified in the two studies—three months and one month, respectively—may reflect differences in patient population heterogeneity and treatment protocols (their study included patients who received RT before immunotherapy, as well as those who underwent immunotherapy as second-line therapy). To our knowledge, this is the first study to propose the concept of IRI in patients with advanced ESCC who received first-line CIT. By comparing survival outcomes between the IRI >1 month and IRI ≤1 month groups, we demonstrate for the first time that a shorter IRI (≤1 month) is associated with significantly prolonged OS.

In terms of safety, hematologic toxicity was comparable between the CI-RT and CIT cohorts, and no significant difference in the rate of grade ≥3 AEs was observed between the two cohorts. The risk of esophagitis was higher in the CI-RT cohort, which is consistent with findings from prior studies;^11^,^20^ however, the majority of AEs were grades 1-2. In addition, one patient in the CIT cohort developed treatment-related pneumonitis, which, despite aggressive symptomatic management, resulted in a fatal AE of Grade-V.

### Limitations:

Our study has several limitations. First, the limited cohort size restricted the feasibility of conducting subgroup analyses of patients receiving concurrent RT and immunotherapy when investigating the optimal timing of RT intervention. Second, as this was a retrospective study, the RT protocols were developed by the treating radiation oncologists and lacked a strict and standardized protocol, which may introduce selection bias. However, our real-world study data not only provide important guidance for current clinical practice but also lay a solid foundation for future prospective research. Next, we plan to collaborate with other centers to expand the sample size and further investigate the effects of concurrent RT and immunotherapy, as well as the impact of RT dose on treatment efficacy.

## CONCLUSIONS

Adding RT to first-line CIT for treating patients with advanced ESCC improves patient prognosis with a favorable safety profile. Patients with oligometastasis and those with “IRI ≤1 month” may derive a more pronounced survival benefit from this combined treatment approach. However, these findings need to be evaluated in future prospective studies with larger cohorts.
